# Introgression of “*QTL‐hotspot*” region enhances drought tolerance and grain yield in three elite chickpea cultivars

**DOI:** 10.1002/tpg2.20076

**Published:** 2021-01-22

**Authors:** Chellapilla Bharadwaj, Shailesh Tripathi, Khela R. Soren, Mahendar Thudi, Rajesh K. Singh, Seema Sheoran, Manish Roorkiwal, Basavanagouda Siddanagouda Patil, Annapurna Chitikineni, Ramesh Palakurthi, Anilkumar Vemula, Abhishek Rathore, Yogesh Kumar, Sushil K. Chaturvedi, Biswajit Mondal, Pichandampalayam Subramaniam Shanmugavadivel, Avinash K. Srivastava, Girish P. Dixit, Narendra P. Singh, Rajeev K. Varshney

**Affiliations:** ^1^ Division of Genetics ICAR–Indian Agricultural Research Institute (ICAR–IARI) New Delhi Delhi 110012 India; ^2^ ICAR–Indian Institute of Pulses Research (ICAR–IIPR) Kanpur Uttar Pradesh 208024 India; ^3^ Center of Excellence in Genomics & Systems Biology International Crops Research Institute for the Semi‐Arid Tropics (ICRISAT) Patancheru Telangana 502324 India; ^4^ Present address: ICAR–Indian Institute of Maize Research (ICAR–IIMR) PAU campus Ludhiana Punjab 141004 India; ^5^ Division of Genetics ICAR–IARI Regional Research Centre for Improvement of Pulses Dharwad Karnataka 580005 India; ^6^ Present address: Rani Lakshmi Bai Central Agricultural University Jhansi Uttar Pradesh 284003 India; ^7^ ICAR–All India Coordinated Research Project on Chickpea (AICRP‐Chickpea), ICAR–IIPR Kanpur Uttar Pradesh India

## Abstract

With an aim of enhancing drought tolerance using a marker‐assisted backcrossing (MABC) approach, we introgressed the “*QTL‐hotspot*” region from ICC 4958 accession that harbors quantitative trait loci (QTLs) for several drought‐tolerance related traits into three elite Indian chickpea (*Cicer arietinum* L.) cultivars: Pusa 372, Pusa 362, and DCP 92‐3. Of eight simple sequence repeat (SSR) markers in the *QTL‐hotspot* region, two to three polymorphic markers were used for foreground selection with respective cross‐combinations. A total of 47, 53, and 46 SSRs were used for background selection in case of introgression lines (ILs) developed in genetic backgrounds of Pusa 372, Pusa 362, and DCP 92‐3, respectively. In total, 61 ILs (20 BC_3_F_3_ in Pusa 372; 20 BC_2_F_3_ in Pusa 362, and 21 BC_3_F_3_ in DCP 92‐3), with >90% recurrent parent genome recovery were developed. Six improved lines in different genetic backgrounds (e.g. BGM 10216 in Pusa 372; BG 3097 and BG 4005 in Pusa 362; IPC(L4‐14), IPC(L4‐16), and IPC(L19‐1) in DCP 92‐3) showed better performance than their respective recurrent parents. BGM 10216, with 16% yield gain over Pusa 372, has been released as Pusa Chickpea 10216 by the Central Sub‐Committees on Crop Standards, Notification and Release of Varieties of Agricultural Crops, Ministry of Agriculture and Farmers Welfare, Government of India, for commercial cultivation in India. In summary, this study reports introgression of the *QTL‐hotspot* for enhancing yield under rainfed conditions, development of several introgression lines, and release of Pusa Chickpea 10216 developed through molecular breeding in India.

## Core Ideas

- Sixty‐one backcross progenies with >90% recurrent parent genome recovery were developed.
- Six superior lines with enhanced drought tolerance and yield performance were nominated for national yield trials in India.
- Pusa Chickpea 10216, the first molecular breeding variety for drought tolerance, was released in India.

**Abbreviations**
100SDW: 100‐seed weight
AICRP: All‐India Coordinated Research Project
AVT: advanced varietal trial
DF: days to 50% flowering
DM: days to maturity
DTIL: drought tolerance introgression line
HI: harvest index
IARI: Indian Agricultural Research Institute
ICAR: Indian Council of Agricultural Research
ICRISAT: International Crops Research Institute for the Semi‐Arid Tropics
IIPR: Indian Institute of Pulses Research
IL: introgression line
MABC: marker‐assisted backcrossing
PHT: plant height
PPP: pods per plant
QTLs: quantitative trait loci
RDW: root dry weight
RL: root length
RLD: root length density
RPG: recurrent parent genome
RSA: root surface area
SNP: single nucleotide polymorphism
SRL: specific root length
SSR: simple sequence repeat
YLD: seed yield per plot.

## 1 INTRODUCTION

Chickpea (*Cicer arietinum* L.) is a cool‐season food legume cultivated on residual soil moisture in southern Asia and sub‐Saharan Africa. Being a rich source of protein, fiber, and other mineral nutrients, it is important for global nutritional and food security. Southeastern Turkey is considered the center of origin of chickpea and after its domestication in the Middle East, chickpea has migrated to the Mediterranean region, India, and Ethiopia (Ladizinsky, 1975; van der Maesen, 1987). Further, in recent years, an increase in cultivated area (17.81 million ha) and production (17.19 million tonnes) has been evidenced (FAOSTAT, 2019). In the case of India, the largest producer and consumer, chickpea production increased from 3.86 to 11.23 million tonnes between 2000–2001 to 2017–2018 (Dixit, Srivastava, & Singh, 2019). Madhya Pradesh, Maharashtra, Rajasthan, Karnataka, and Andhra Pradesh are major chickpea growing states in India. Abiotic and biotic stresses hamper chickpea production, especially terminal drought or end‐season drought has alone been reported to cause >50% yield losses (Ahmad, Gaur, & Croser, 2005). Globally, the frequency of occurrence and severity of drought is predicted to increase in the climate change scenario (Carrão, Naumann, & Barbosa, 2018). In India, during the last five decades, drought has been reported to occur at least once in every 3 yr (Mishra, Singh, & Desai, 2009, United Nations Office for Disaster Risk Reduction, 2009). In the case of central and southern India, where the occurrence of drought is more frequent, to mitigate the adverse effects of drought, the chickpea research community has leveraged drought escape and drought avoidance mechanisms (Berger, Palta, & Vadez, 2016; Gaur et al., 2019). According to the Vision 2050 document of Indian Council of Agricultural Research (ICAR)–Indian Institute of Pulses Research (IIPR), about 16–17.5 million tonnes of chickpea needs to be produced by 2050 from an area of about 10.5 million ha with average productivity of 1.5–1.7 t ha^−1^ (Dixit et al., 2019; https://iipr.icar.gov.in/pdf/vision_250715.pdf). However, to achieve this self‐sufficiency, deeper understanding of genetics of drought tolerance and developing the drought‐tolerant chickpea cultivars is required.

Drought being a complex trait, earlier efforts to understand the genetics of the trait had a major focus on morphological, biochemical, and physiological traits associated that contribute to drought tolerance (Gunes et al., 2006; Mafakheri, Siosemardeh, Bahramnejad, Struik, & Sohrabi, 2010; Purushothaman, Krishnamurthy, Upadhyaya, Vadez, & Varshney, 2016; Upadhyaya et al., 2012; Varshney, Tuberosa, & Tardieu, 2018). The role of traits like transpiration efficiency and carban isotope discrimination in mitigating terminal drought was also reported (Kashiwagi et al., 2005). In a recent study, conservative water use benefitting seed yield of chickpea under terminal drought conditions has been reported (Pang, Turner, Du, Colmer, & Siddique, 2017). The traits of relevance and scope for improving yield under drought has also been presented (Kashiwagi et al., 2013, 2015). Root traits, especially root length density (RLD [g cm^−3^]) was reported to play a key role in mitigating the effects of drought by studying the root system architecture and its plasticity in chickpea germplasm lines (Kashiwagi, Krishnamurthy, Gaur, Chandra, & Upadhyaya, 2008). Anatomical studies on chickpea root system showed moderate xylem passage (number of xylem vessels × average vessel diameter) per root indicating that chickpea is capable of absorbing water moderately under drought conditions (Purushothaman et al., 2013). Besides profuse RLD at surface soil depths, root dry weight at deeper soil layers and high root/shoot ratio was proposed to be the best selection strategy for yield under terminal drought conditions in chickpea (Purushothaman, Krishnamurthy, Upadhyaya, Vadez, & Varshney, 2017).

During the last two decades, the genetic dissection of several complex traits has greatly improved as a result of the genomics revolution (Roorkiwal et al., 2020). Draft genome (Varshney et al., 2013b) and sequencing of >400 germplasm accessions (Thudi et al., 2016a, 2016b; Varshney et al., 2019) provided several thousands of SSR markers and millions of single nucleotide polymorphism (SNP) markers for trait dissection and their use in chickpea breeding programs. For instance, several high‐density genetic maps have been developed (Deokar, Sagi, Daba, & Tar'an, 2019; Roorkiwal et al., 2018; Thudi et al., 2011) and several traits, including drought tolerance, have been investigated at molecular level (Pushpavalli et al., 2015; Sab et al., 2020; Sivasakthi et al., 2018; Vadez et al., 2012; Varshney et al., 2014a). Efforts were also made to understand the genes involved in drought tolerance through transcriptomic studies (Mantri, Ford, Coram, & Pang, 2007; Mashaki et al., 2018; Varshney et al., 2009). Although several studies reported QTLs for drought‐tolerance related traits (Hamwieh, Imtiaz, & Malhotra, 2013; Rehman, Malhotra, Bett, Bueckert, & Warkentin, 2011), the *QTL‐hotspot* genomic region reported by Varshney et al. (2014a) explained the major phenotypic variation (>50%) for drought tolerance. Further, using different genotyping approaches the *QTL‐hotspot* was fine mapped and genes present in this genomic region have been reported (Jaganathan et al., 2015; Kale et al., 2015). In addition, genome‐wide markers associated with drought tolerance and mid‐reproductive stage canopy temperature depression were also reported (Li et al., 2018; Purushothaman et al., 2015; Thudi et al., 2014a; Varshney et al., 2019).

Marker‐assisted backcrossing (MABC) breeding has been successfully deployed for improving biotic, abiotic, and nutrition related traits in several crops (Kang et al., 2019; Oladosu et al., 2020; Prasanna et al., 2020; Singh, Sharma, Varshney, Sharma, & Singh, 2008; Thudi et al., 2014b) including legumes (Bohra, Saxena, Varshney, & Saxena, 2020; Pandey et al., 2020; Roorkiwal et al., 2020; Varshney, 2016). In the case of chickpea, superior lines with enhanced drought tolerance (Varshney et al., 2013a) and resistance to Fusarium wilt and Ascochyta blight have been reported using the MABC approach (Mannur et al., 2019; Pratap et al., 2017; Varshney et al., 2014b). Fusarium wilt resistant variety MABC‐WR‐SA1, also called Super Annigeri 1, with 7% more yield potential than Annigeri 1 (Mannur et al., 2019) has also been released for commercial cultivation in India by Central Sub‐Committees on Crop Standards, Notification and Release of Varieties of Agricultural Crops, Ministry of Agriculture and Farmers Welfare, Government of India.

The present study reports the development of 61 superior lines with higher seed yield and enhanced drought tolerance in three genetic backgrounds, namely Pusa 372 and Pusa 362 at ICAR–Indian Agricultural Research Institute (IARI) and DCP 92‐3 at ICAR–IIPR in collaboration with International Crops Research Institute for the Semi‐Arid Tropics (ICRISAT). We also report the release of Pusa Chickpea 10216, a molecular breeding variety with enhanced drought tolerance and increased yield under rainfed conditions in India. Our study confirms that the introgression of the *QTL‐hotspot* region enhances drought tolerance and yield irrespective of the genetic backgrounds.

**Core Ideas**

- Sixty‐one backcross progenies with >90% recurrent parent genome recovery were developed.
- Six superior lines with enhanced drought tolerance and yield performance were nominated for national yield trials in India.
- Pusa Chickpea 10216, the first molecular breeding variety for drought tolerance, was released in India.

## 2 MATERIALS AND METHODS

### 2.1 Selection of donor and recipient parents

With an aim of enhancing the drought tolerance in elite cultivars, the MABC approach was adopted independently at ICAR–IARI and ICAR–IIPR. For achieving this, ICC 4958 (http://oar.icrisat.org/540/1/PMD_33.pdf) was used as donor parent to introgress the *QTL‐hotspot* into three elite cultivars namely Pusa 372, Pusa 362, and DCP 92‐3. Donor ICC 4958 is a landrace collected from Jabalpur, Madhya Pradesh, India, in 1973. It is used as a drought‐tolerant donor parent that produces high yields in short‐duration, terminal‐drought environments regions.

### 2.2 Markers for foreground and background selection

Initially, eight SSR markers (TAA170, ICCM0249, GA24, STMS11, NCPGR21, NCPGR127, GA11, and TR11) in the *QTL‐hotspot* region (Varshney et al., 2014a) were used for parental polymorphism analysis on three selected recipient parents (Pusa 362, Pusa 372, and DCP 92‐3) and donor parent (ICC 4958) for possible use in foreground selection. Polymerase chain reaction (PCR) for all markers as performed in 5‐μl reaction volume as described earlier (Varshney et al., 2013a). The PCR amplicons were either resolved on 1.2% agarose gel or using ABI 3730 (Applied Biosystems). For foreground selection, polymorphic markers with donor and recipient cross‐combinations from the *QTL‐hotspot* genomic region were used (Supplemental Table S1). Based on earlier studies, a set of 346 highly polymorphic SSR markers (Nayak et al., 2010; Thudi et al., 2011; Varshney et al., 2014a) were tested for parental polymorphism among donor and recipient parents for possible use in background selection at the Center of Excellence in Genomics and Systems Biology (cegsb.icrisat.org), ICRISAT. Polymorphic markers identified for each donor and recipient parent cross‐combination were used for background selection (Supplemental Table S2). The percentage of recurrent parent genome (RPG) recovery was calculated as described earlier (Mannur et al., 2019).

### 2.3 Introgression of the *QTL‐hotspot* genomic region into three genetic backgrounds

**FIGURE 1:**
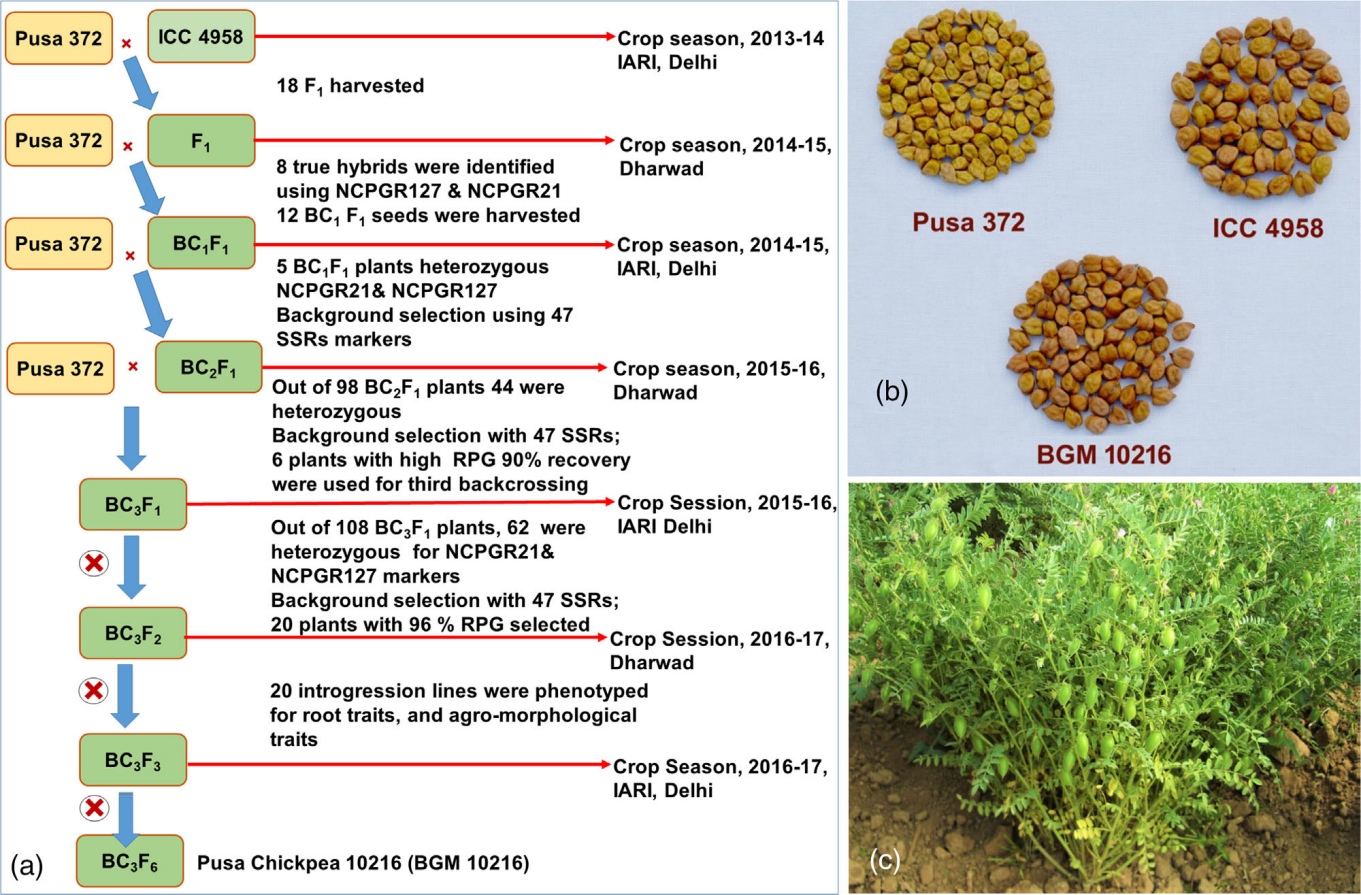
Marker‐assisted backcrossing (MABC) scheme for introgression of the “*QTL‐hotspot*” genomic region in Pusa 372 at ICAR–IARI. (a) Details of the MABC scheme adopted, (b) MABC line showing increased seed size, (c) One introgression line in the field

**FIGURE 2:**
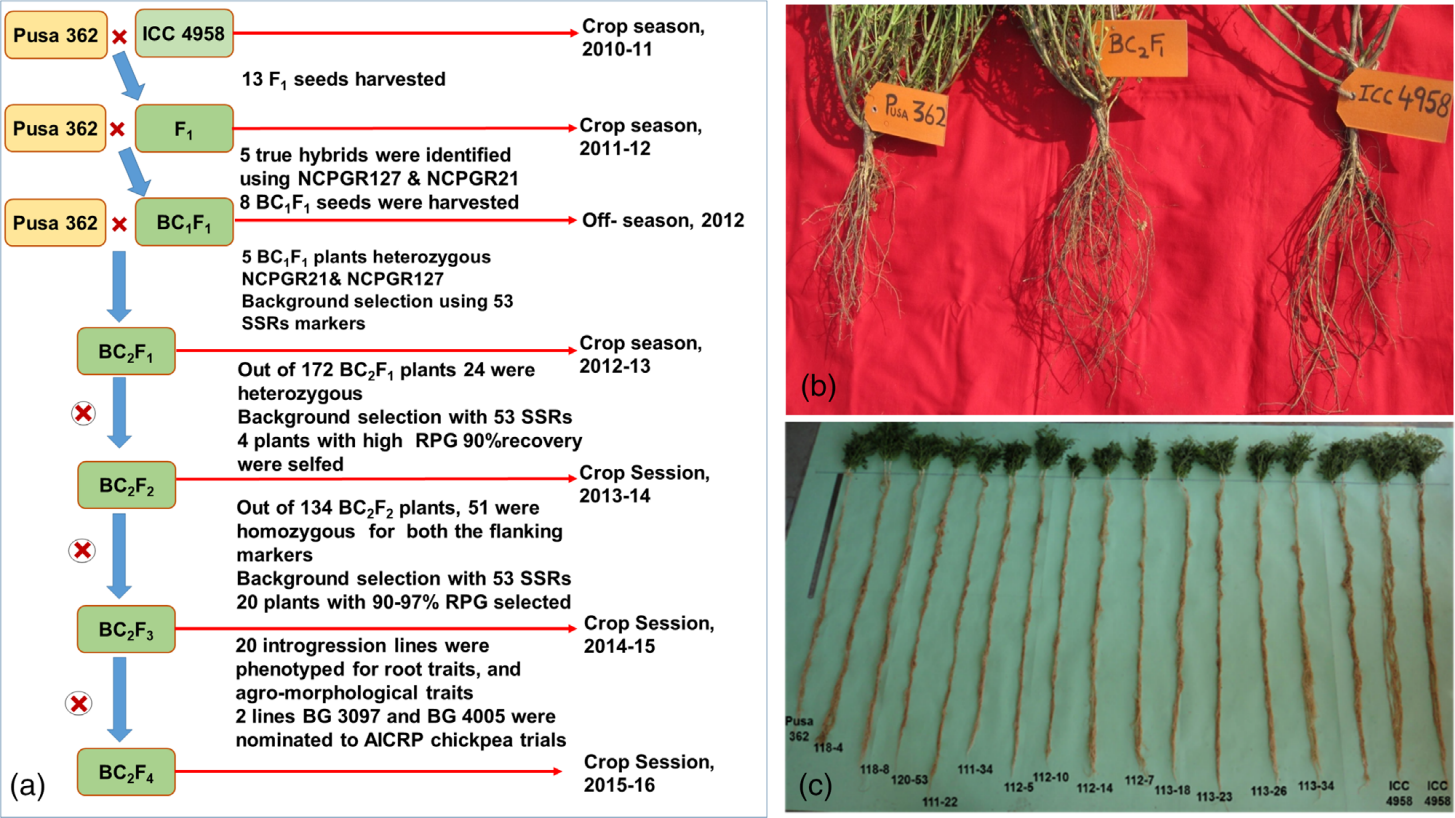
Marker‐assisted backcrossing (MABC) scheme for introgression of the “*QTL‐hotspot*” genomic region in Pusa 362 at ICAR–IARI. (a) Details of the MABC scheme adopted, (b) One MABC line showing higher root biomass compared with the recurrent (Pusa 362) and donor (ICC 4958) genotypes, (c) MABC lines showing higher root length compared with the recurrent (the left most) and donor (the last two lines on right side) parents

**FIGURE 3:**
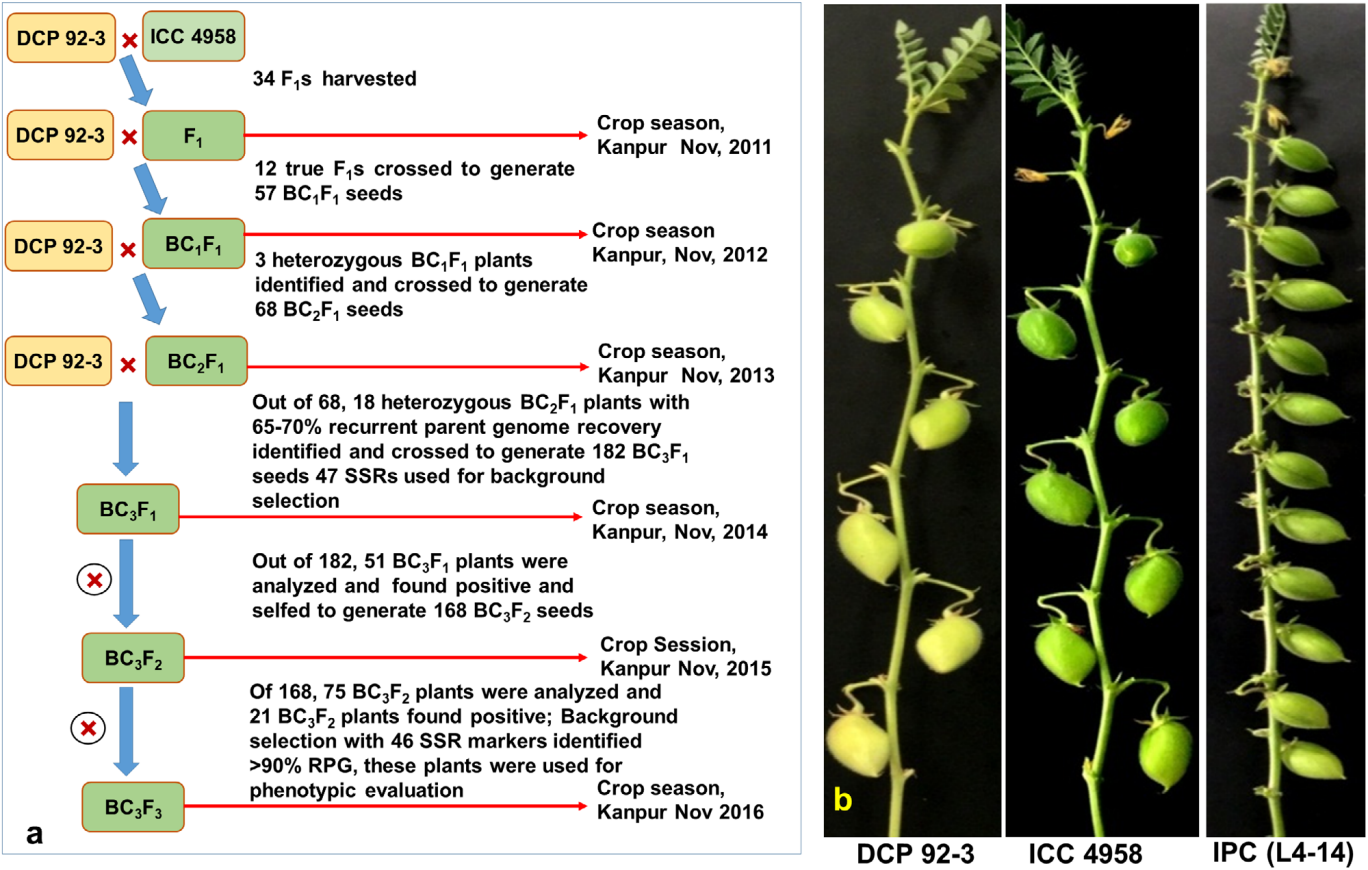
Marker ‐assisted backcrossing (MABC) scheme for introgression of the “*QTL‐hotspot*” genomic region in DCP 92‐3 at ICAR–IIPR. (a) Details of the MABC scheme adopted, (b) One MABC line (IPC[L4‐14]), the right most line) showing higher number of pods compared with the recurrent (DCP 92‐3) and donor (ICC 4958) genotypes

The *QTL‐hotspot* region was introgressed independently into the chosen recipient parents employing the MABC approach. At ICAR–IARI, a set of 20 BC_3_F_3_ lines were developed by crossing Pusa 372 with ICC 4958 followed by three backcrossing and two subsequent selfing generations (Figure 1). Similarly, 20 BC_2_F_3_ lines were developed by crossing Pusa 362 with ICC 4958, also at ICAR–IARI followed by two backcrossing and two subsequent selfing generations (Figure 2). Similarly, 21 BC_3_F_3_ lines were developed at ICAR–IIPR by crossing DCP 92‐3 with ICC 4958 followed by three backcrossing and two subsequent selfing generations (Figure 3).

### 2.4 Phenotyping of MABC introgression lines

A set of selected 20 BC_3_F_3_ lines in the genetic background of Pusa 372 along with the donor and recipient parents were evaluated in two‐row plots of 2‐m row length in the rain‐out shelter for the rainfed experiment. The ILs were phenotyped for yield‐contributing traits, that is, plant height (PHT [cm]), days to 50% flowering (DF, (d)), days to maturity (DM, (d)), pods per plant (PPP), 100‐seed weight (100SDW [g]), and seed yield per plot (YLD [g m^−2^]) at the experimental farm of ICAR–IARI, New Delhi (28.6139° N, 77.2090° E) during the year 2016–2017. The data were taken on five single plants and averaged. Soil moisture was 40% available at pod formation. Based on the yield performance, the superior line Pusa 372_IL12 designated as BGM 10216 was nominated for national trials under ICAR–All‐India Coordinated Research Project (AICRP) on Chickpea. The superior line was evaluated along with the donor and recipient parents in different locations in advanced varietal trial (AVT) 1 and AVT2 during 2017–2018 and 2018–2019, respectively.

Another set of 20 BC_2_F_3_ lines in the genetic background of Pusa 362 along with donor (ICC 4958) and recipient parents were phenotyped for root traits, phenological traits, and yield‐related traits at the experimental farm of ICAR–IARI, New Delhi, during the year 2015–2016. Randomized complete block design with three replications under rainfed environment was conducted following all recommended cultural and agronomic practices. Each genotype was planted in a four ‐row plot of 5‐m length with 10 and 30 cm between plants and rows, respectively. Root traits, that is, root dry weight (RDW [g]), root length (RL [cm]), root surface area (RSA [mm^2^]), and specific root length (SRL [cm g^−1^]), were recorded as described earlier (Varshney et al., 2014a). Phenological traits and yield‐related traits include PHT, DF, DM, PPP, biomass (g), harvest index (%), and YLD.

A total of 21 BC_3_F_3_ lines along with recurrent (DCP 92‐3) and donor (ICC 4958) parents were phenotyped under water‐stress conditions at ICAR–IIPR, Kanpur, India (26°26′59.7228′′ N, 80° 19′54.7356′′ E) during November to April 2015–2016. Random block design with plot size 4 × 0.3 m (1.2 m^2^) in three replications and four blocks within replication were adopted for conducting the experiment. Approximately 40 seeds were maintained per row and recommended crop management practices were followed at the experimental location. Data were collected on plot basis on DF, DM, primary branch, secondary branch, tertiary branch, YLD, PPP, PHT, and 100SDW. The three best performing BC_3_F_3_ lines—IPC(L4‐14), IPC(L4‐16), and IPC(L19‐1)—were selected based on yield and other yield‐attributing agronomic traits under moisture stress and further phenotyped under water‐stress and non‐stress conditions during November to April 2016–2017. The moisture stress was maintained depending on the rainfall throughout the cropping season and grain yield data was recorded for water stress and nonstress conditions and lines with lower drought susceptible index were identified.

### 2.5 Statistical analysis

Family‐wise ANOVA was carried out using PROC GLM (SAS Institute, 2016) considering replication, MABC lines as fixed. Least‐square means were calculated from analysis of variance.

## 3 RESULTS

### 3.1 Marker polymorphism, foreground, and background selection

Among eight SSR markers in the *QTL‐hotspot* region tested for polymorphism, two markers (NCPGR21 and NCPGR127) were found polymorphic in the case of Pusa 372 × ICC 4958 and Pusa 362 × ICC 4958 combinations (Supplemental Table S1). Hence these two markers (NCPGR21 and NCPGR127) were used for confirmation of hybrids in the F_1_ generation and foreground selection in subsequent generations in the case of Pusa 362 × ICC 4958 (BC_1_F_1_, BC_2_F_1_) and Pusa 372 × ICC 4958 (BC_1_F_1_, BC_2_F_1_ and BC_3_F_1_) crosses. In the case of DCP 92‐3 × ICC 4958 combination, three polymorphic SSR markers (TAA170, NCPGR21, and TR11) were used for confirmation of hybrids in the F_1_ generation and foreground selection in subsequent generations (BC_1_F_1_, BC_2_F_1_, and BC_3_F_1_) (Supplemental Table S1).

For background selection, among the 346 SSR markers tested on the selected donor and recipient parents, 129 markers showed informative polymorphisms that can be used for background selection. Among the polymorphic markers, six to eight markers per linkage group that were equally distributed across the chickpea genome were selected and used for background selection. Thus, in total, 47, 53, and 46 polymorphic markers were used for background selection for Pusa 372 × ICC 4958, Pusa 362 × ICC 4958, and DCP 92‐3 × ICC 4958 crosses, respectively (Supplemental Table S2).

### 3.2 Introgression of the *QTL‐hotspot* genomic region in elite chickpea cultivars

#### 3.2.1 Introgression of the *QTL‐hotspot* genomic region in Pusa 372

A total of 18 F_1_ seeds were harvested by crossing Pusa 372 × ICC 4958 in the postrainy season of 2013–2014 at the ICAR–IARI farm, New Delhi. These seeds were grown in the off‐season nursery at the ICAR–IARI regional station, Dharwad in the rainy season of 2014–2015, and eight heterozygous F_1_ plants identified using NCPGR127 and NCPGR21 markers were backcrossed with the recurrent parent and 12 BC_1_F_1_ seeds were obtained. Of these 12 BC_1_F_1_ seeds, based on foreground selection using NCPGR127 and NCPGR21, five BC_1_F_1_ plants (with higher genome recovery using 47 SSR markers) were selected and then used for the second cycle of backcrossing in 2014–2015 at ICAR–IARI farm, New Delhi. Subsequently, approximately 98 BC_2_F_1_ seeds were harvested. From the 98 BC_2_F_1_ plants, 44 heterozygous plants selected using foreground markers were screened with 47 SSR markers for background selection (Supplemental Table S2). As a result, six BC_2_F_1_ plants with high RPG recovery (90%) were used for a third backcrossing at ICAR–IARI regional station, Dharwad, to generate 108 BC_3_F_1_ (Figure 1a). Upon background selection using 47 SSR markers, 20 BC_3_F_1_ plants showing 96% of RPG recovery were selected for selfing. Finally, after two generations of selfing, 20 best BC_3_F_3_ plants were evaluated based on foreground and background selection, as well as agronomic performance and evaluated at ICAR–IARI Delhi. The best‐performing line, BGM 10216, was nominated for ICAR–AICRP on Chickpea trials.

#### 3.2.2 Introgression of the *QTL‐hotspot* genomic region in Pusa 362 variety

In the case of introgression of the *QTL‐hotspot* genomic region in Pusa 362 variety, 13 F_1_ seeds were harvested after crossing Pusa 362 with ICC 4958 at ICAR–IARI farm, New Delhi. Five true F_1_ plants identified using NCPGR127 and NCPGR21 markers were backcrossed with the recurrent parent, and eight BC_1_F_1_ seeds were obtained in a greenhouse during the off‐season in 2012. Of these eight BC_1_F_1_, five BC_1_F_1_ plants (with higher genome recovery using 53 SSR markers) were selected and then used for a second cycle of backcrossing during crop season 2012–2013. Subsequently, approximately 172 BC_2_F_1_ seeds were harvested. Of the 172 BC_1_F_1_, 24 heterozygous plants selected based on foreground selection were screened with 53 SSR markers for background selection (Supplemental Table S2). As a result, four BC_2_F_1_ plants with high RPG recovery (90%) were selfed to generate 134 BC_2_F_2_ plants. Of these 134 BC_2_F_2_ plants, 51 were found homozygous for both flanking markers (NCPGR127 and NCPGR21). Of 51 homozygous plants, 20 plants with 90–97% RPG were selected to raise BC_2_F_3_ plants (Figure 2a). Based on agronomic performance, BG 3097 line was nominated for ICAR–AICRP on Chickpea trials.

#### 3.2.3 Introgression of the *QTL‐hotspot* genomic region in DCP 92‐3 variety

DCP 92‐3 variety was targeted for introgressing the *QTL‐hotspot* genomic region at ICAR–IIPR, Kanpur. Of 34 F_1_ plants derived from DCP 92‐3 × ICC 4958 cross (crop season 2011–2012), 12 plants confirmed as true hybrids with foreground markers (TAA170, NCPGR21, and TR11) were used for making the first backcross, and 57 BC_1_F_1_ seeds were harvested during crop season 2012–2013 (Figure 3a). Based on foreground selection, three BC_1_F_1_ plants were selected and backcrossed with recurrent parent to generate 68 BC_2_F_1_ seeds. Of the 68 BC_2_F_1_ seeds harvested during 2013–2014, 18 BC_2_F_1_ plants found positive for the *QTL‐hotspot* alleles were selected for background selection. The RPG recovery varied between 65 and 70% among the 18 BC_2_F_1_ plants using 46 markers. The selected 18 BC_2_F_1_ plants were subjected to one more round of backcrossing and, subsequently, 182 BC_3_F_1_ seeds were harvested during crop season 2014–2015. After foreground selection, 51 BC_3_F_1_ plants were found positive and selfed to generate 168 BC_3_F_2_ seeds. Of the 168 BC_3_F_2_ plants, 75 BC_3_F_2_ plants were analyzed with foreground markers and 21 BC_3_F_2_ plants were found positive. The RPG recovery of 21 BC_3_F_2_ plants ranged from 89 to 94% using 46 SSR markers (Supplemental Table S2).

### 3.3 Phenotypic evaluation of introgression lines

#### 3.3.1 Performance of Pusa 372 ILs

Twenty ILs (BC_3_F_3_ lines) along with donor (ICC 4958) and recurrent parent (Pusa 372) were evaluated for different morphological, phenological, and yield‐related traits under rainfed conditions during 2016–2017 at ICAR–IARI fields. Analysis of variance for the traits studied indicated a significant difference among the ILs (Supplemental Table S3). Although ILs differed significantly for DM, PPP, and YLD, they did not differ significantly from the recurrent parent for PHT and DF. Among 20 ILs, two ILs (Pusa 372_IL12 and Pusa 372_IL17) recorded maximum yield (22%) over the recurrent parent (Supplemental Table S4). The increased yield is a result of the increased number of PPP (Figure 1b). Further, it was also noted that, all ILs recorded higher seed yield than both recurrent as well as donor parents. Among the ILs, 100SDW ranged from 20.19 (Pusa 372_IL05) to 24.32 g (Pusa 372_IL01) with an average of 22.7 g. Nevertheless, none of the ILs had 100SDW greater than the donor parent (Figure 1c). The results indicate that, morphologically, the ILs were like the recurrent parent, Pusa 372. Days to maturaty among the ILs ranged from 105 (Pusa 372_IL15) to 126 d (Pusa 372_IL12), and the number of PPP among the ILs varied from 52 to 227 with an average of 177.64 pods.

Under the ICAR–AICRP on Chickpea, a special trial known as ‘drought tolerance introgression lines’ (DTILs) evaluated the ILs developed by introgressing the *QTL‐hotspot* into different genetic backgrounds. Among the best performing lines, one of the lines, Pusa 372_IL12 designated as BGM 10216, was evaluated in six and five locations during crop season 2017–2018 and 2018–2019 in AVT1 and AVT2, respectively. BGM 10216 outperformed Pusa 372 in four out of six locations in AVT1 and all five locations in AVT2 trials (Figure 1d). In overall performance, BGM 10216 recorded a highest mean yield 1,475 kg ha^−1^ with a potential 2,575 kg ha^−1^ under drought‐stress conditions over the recurrent parent Pusa 372 with mean yield 1,272 kg ha^−1^. It recorded an overall weighted percentage increase over the mean of 16% with 8% in AVT1 (Table 1) and 30% in AVT2 (Table 2). Advanced varietal trial data also indicated that it is an early flowering (50–55 d) and early maturing variety (106 d).

**TABLE 1:** Comparison of yield (kg ha^−1^) performance of BGM 10216, one introgression line developed in the genetic background of Pusa 372, with the recurrent genotype at six locations in the Advanced Varietal Trials−1 of ICAR–All India Coordinated Research Project on Chickpea conducted during 2017–2018 (Source: AICRP Chickpea Annual Report 2017–2018)

Entries	Vijayapura	Dharwad	Rahuri	Badnapur	Arnej	Gulbarga	Mean	Frequency^a^
BGM 10216	2,574	1,033	1,854	1,141	1,302	1,725	1,605	4/6
Pusa 372 (recurrent parent)	2,831	935	1,724	1,207	1,110	1,117	1,487	2/6
Critical difference at 5%	NS^†^	275	373	217	141	385	–	–
CV (%)	10.2	15.7	13	11.9	7.4	17	–	–
State avg. yield (kg ha^−1^)	591	591	731	731	1,227	591	–	–

^a^ Frequency, the ratio of number locations in which the introgression line performs higher than the recurrent parent to the total number of locations evaluated.

^†^NS, nonsignificant at 5% level of significance.

**TABLE 2:** Comparison of yield (kg ha^−1^) performance of BGM 10216, one introgression line developed in the genetic background of Pusa 372, with the recurrent genotype at five locations in the Advanced Varietal Trials−2 of ICAR–All India Coordinated Research Project on Chickpea conducted during 2018–2019 (Source: AICRP Chickpea Annual Report 2018–2019)

Entries	Pedigree	Kalaburagi	Coimbatore	Vijayapura	Badnapur	Rahuri	Mean	Frequency^a^
BGM 10216	(Pusa 372 × ICC 4958) × 2 × Pusa 372)	2,321	846	1,065	1,396	961	1,318	5/5
Pusa 372 (recurrent parent)	–	2,101	706	331	1,225	708	1,014	0/5
Critical difference at 5%	–	346	67	223	296	245	–	–
CV (%)	–	10.6	5.1	16.2	1,408	19.9	–	–
State avg. yield (kg ha^−1^)	–	559	742	559	782	782	–	–

^a^ Frequency, the ratio of number locations in which the introgression line performs higher than the recurrent parent to the total number of locations evaluated.

#### 3.3.2 Performance of Pusa 362 ILs

Twenty BC_2_F_3_ lines along with donor and recurrent parent (Pusa 362) were evaluated for root traits and phenological and yield‐related traits. Except for the RDW trait, the ILs differed significantly for all other root traits under rainfed conditions (Supplemental Table S5). Some of the ILs had RL and RLD values greater than both donor and recurrent parents (Figure 2b and 2c). The mean RDW in ILs ranged from 0.45 (Pusa 362_IL18) to 1.4 g (Pusa 362_IL06) (Supplemental Table S6) with the average being 0.96 g. Seventeen out of 20 ILs had higher RDW than the donor parent ICC 4958. Three ILs—Pusa 362_IL06, Pusa 362_IL09, and Pusa 362_IL04—were similar to recurrent parent Pusa 362 for RDW. The SRL in the ILs varied from 88.59 (Pusa 362_IL06) to 244.99 cm g^−1^ (Pusa 362_IL18) with an average of 141.9 cm g^−1^. Pusa 362_IL18 and Pusa 362_IL19 were significantly superior to the donor parent in SRL. Although no significant difference among replications was noted, we observed a significant difference among the ILs for DM, PPP, and biomass. Further, the ILs also differed significantly from the parents for PPP, 100SDW, and harvest index (Supplemental Table S7). The average PHT varied from 33.9 (Pusa 362_IL17) to 44.3 cm (Pusa 362_IL20) with an average of 38.8 cm. The results indicate that morphologically, the ILs were like the recurrent parent Pusa 362. There was significant variation among the ILs for PPP, which ranged from 33 in Pusa 362_IL14 to 50 in Pusa 362_IL6 and Pusa 362_IL19 with over all mean of 41.81 pods per plant (Supplemental Table S8). The PPP in recurrent parent Pusa 362 was 48. Biomass per plot ranged from 415.6 (Pusa 362_IL07) to 800 g (Pusa 362_IL05) with mean of 561.58 g. Seed yield per plot ranged from 324 (Pusa 362_IL13) to 568.7 g (Pusa 362_IL05) with mean seed yield 418.8 g. The recurrent parent Pusa 362 yielded 521.33 g while the donor parent ICC 4958 gave a yield of 276 g. Pusa 362_IL05 was the best IL, which gave significantly higher yield than Pusa 362 under rainfed condition.

Among the best performing lines, one of the lines, designated as BG 3097 (IL05), was evaluated in six locations during crop season 2017–2018 in AVT1 and at four locations during 2018–2019 in AVT2. BG 3097 recorded an overall weighted mean yield advantage of 8.0% over recurrent parent Pusa 362 in DTIL trial across 10 locations under AICRP over two consecutive years (AVT1 & AVT2; Table 3 and 4). Overall, BG 3097 showed mean yield superiority of 12.4% over national check JG 16 and 3.5% over JAKI 9218 under drought conditions. Overall, BG 3097 recorded a weighted mean yield of 1,374 kg ha^−1^ with a potential 1,902 kg ha^−1^ under drought‐stress conditions over the recurrent parent Pusa 362, which recorded weighted mean yield of 1,272 kg ha^−1^.

**TABLE 3:** Comparison of yield (kg ha^−1^) performance of BG 3097, one introgression line developed in the genetic background of Pusa 362, with the recurrent genotype at six locations in the Advanced Varietal Trials−1 of ICAR–All India Coordinated Research Project on Chickpea conducted during 2017–2018 (Source: AICRP Chickpea Annual Report of 2017–2018)

Entry	Pedigree	Vijayapura	Dharwad	Rahuri	Badnapur	Arnej	Gulbarga	Mean	Frequency^a^
BG 3097	(Pusa 362 × ICC 4958) × 2 × Pusa 362)−51	2,727	1,094	1,599	1,043	1,281	402	1,285	5/6
Pusa 362 (recurrent parent)	–	2,541	1,211	1,240	898	1,017	402	1,140	1/6
Critical difference at 5%	–	NS	275	373	217	141	385	–	–
CV (%)	–	10.2	15.7	13	11.9	7.4	17	–	–
General mean (kg ha^−1^)	–	2,641	1,235	1,954	1,290	1,343	1,362	–	–
State avg. yield (kg ha^−1^)	–	591	591	731	731	1,227	591	–	–

^a^ Frequency, the ratio of number locations in which the introgression line performs higher than the recurrent parent to the total number of locations evaluated.

**TABLE 4:** Comparison of yield (kg ha^−1^) performance of BG 3097, one introgression line developed in the genetic background of Pusa 362, with the recurrent genotype at four locations in the Advanced Varietal Trials−2 of ICAR–All India Coordinated Research Project on Chickpea conducted during 2018–2019 (Source: AICRP Chickpea Annual Report of 2018–2019)

Entries	Pedigree	Kalaburagi	Coimbatore	Vijayapura	Badnapur	Mean	Frequency^a^
BG 3097	(Pusa 362 × ICC 4958) × 2 × Pusa 362)−51	2,592	635	1,077	1,291	1,399	5/4
Pusa 362 (recurrent parent)	–	2,507	434	1,571	895	1,352	1/4
Critical difference at 5%	–	346	67	223	296	–	–
CV (%)	–	10.6	5.1	16.2	14.8	–	–
General mean (kg ha^−1^)	–	2,299	917	972	1,210	–	–
State avg. yield (kg ha^−1^)	–	559	742	559	782	–	–

^a^ Frequency, the ratio of number locations in which the introgression line performs higher than the recurrent parent to the total number of locations evaluated.

#### 3.3.3 Performance of DCP 92‐3 ILs

A total of 21 ILs (BC_3_F_3_ lines) in the genetic background of DCP 92‐3 were evaluated for different agronomic and yield‐related traits at the ICAR–IIPR, Kanpur, fields. No significant difference in performance was observed among the replications. However, the MABC lines did differ significantly for all traits except primary branches (Supplemental Table S9). Of 21 ILs, 17 ILs showed 100SDW more than the recurrent parent and four ILs—IPC7(20), IPC9(5), IPC19(1), and IPC21(7)‐2—had 100SDW less than the recurrent parent. Except for IPC4(11), IPC4(2), IPC4(24), and IPC4(26) ILs, all other ILs had higher yield than DCP 92‐3. In brief, 16 ILs outperformed ICC 4958 in terms of yield; however, five ILs—IPC4(11), IPC4(18), IPC4(2), IPC4(24), and IPC4(26)—yielded less than the donor parent (Supplemental Table S10). The number of PPP increased significantly in the case of IPC(L4‐14) (Figure 3b). During 2016–2017, three ILs—IPC(L4‐14), IPC(L4‐16), and IPC(L19‐1)—were evaluated under rainfed and irrigated condition along with DCP 92‐3 at ICAR–IIPR, Kanpur.

Three superior ILs—IPC(L4‐14), IPC(L4‐16), and IPC(L19‐1)—having positive alleles of the *QTL‐hotspot* region were nominated for testing in AVT1 trials of ICAR–AICRP on Chickpea during crop season 2018–2019. Although the mean yield of all three lines tested was higher than the mean yield of recurrent parent, the line IPC(L4‐14) recorded 5–38% increase in yield over recurrent parent in four different locations (Table 5). Hence, this line has been selected for further testing for its yield performance in upcoming AVT2 trials during the crop season 2019–2020.

**TABLE 5:** Summary of yield performance of three improved lines in different locations compared with recurrent in Advanced Varietal Trials−1 conducted during 2018–2019

Entries	Pedigree	Kalaburagi	Coimbatore	Vijayapura	Badnapur	Rahuri	Nandyal	Arnej	Sehore	Mean	Frequency^a^
IPC(L4‐14)	(DCP 92‐3 × ICC 4958) × 3 × DCP 92‐3	2,272	879	1,547	1,304	659	560	1,068	1,311	1,332	4/7
IPC(L4‐16)	(DCP 92‐3 × ICC 4958) × 3 × DCP 92‐3	2,485	446	433	1,156	899	–	625	1,103	1,084	1/6
IPC(L19‐1)	(DCP 92‐3 × ICC 4958) × 3 × DCP 92‐3	2,014	611	1,076	1,033	–	–	594	803	1,184	–
DCP 92‐3 (ch)	–	2,311	927	1,202	1,109	–	–	770	1,238	1,387	1/7
General mean (kg ha^−1^)	–	2,299	917	972	1,210	855	953	1,093	1,099	–	–
Critical difference at 5%	–	346	67	223	296	245	162	184	148	–	–
CV (%)	–	10.6	5.1	16.2	14.8	19.9	11.8	11.9	9.5	–	–
State avg. yield (kg ha^−1^)	–	559	742	559	782	782	1,051	1,244	1,165	–	–

^a^ Frequency, the ratio of number locations in which the introgression line performs higher than the recurrent parent to the total number of locations evaluated.

## 4 DISCUSSION

In the context of rapid climate changes, development of climate‐resilient varieties is prerequisite to attain sustainable crop production and meet the global food demands. Achieving self‐sufficiency and meeting future demands for food grains may not be possible using conventional breeding approaches alone. To accelerate faster genetic gains and make small‐holder agriculture profitable, the integration of approaches like genomics, phenotyping, and systems modelling and agronomy are essential (Varshney et al., 2018). Recently, the ‘5Gs’ (genome assembly, germplasm characterization, gene function identification, genomic breeding, and gene editing) breeding approach has been proposed for achieving precision and enhancing the crop improvement to meet the future demands of nutritious food (Varshney et al., 2020). Climate changes in recent years increased the occurrence of more severe, longer, and intense droughts in most of the areas in northern and eastern India (Ge, Huang, Xu, Qi, & Liu, 2014). Hence, breeding for drought‐tolerant chickpeas is a priority. This a first study in pulses that reports enhancement of drought tolerance is multiple genetic backgrounds of chickpea as well as release of one drought‐tolerant molecular breeding variety for commercial cultivation using the MABC approach. In the present study, we report development of 61 ILs (20 BC_3_F_3_ ILs in Pusa 372, 20 BC_2_F_3_ ILs in Pusa 362, and 21 BC_3_F_3_ ILs in DCP 92‐3) by introgressing the *QTL‐hotspot* genomic region that harbors QTLs for several drought‐tolerant related traits.

Of eight SSR markers in the *QTL‐hotspot* genomic region, none of the three donor and recipient parent combinations had all eight markers polymorphic. Hence, we used two to three polymorphic SSR markers with respective cross‐combinations for foreground selection at each generation. The low percentage of marker polymorphism is not uncommon in highly self‐pollinated species like chickpea. Three SSRs markers (TAA170, ICCM0249, and STMS11) were used for foreground selection while introgressing the *QTL‐hotspot* genomic region into JG 11 genetic background. Further, based on marker polymorphism, we used 46 to 53 markers for estimating the RPG recovery. In the case of ILs in Pusa 362 background, 90–97% of RPG recovery was observed, hence the third cycle of backcrossing was not taken like the other two MABC programs. Earlier, higher background genome recovery in the second backcross generation was also reported in the case of molecular breeding lines with enhanced Fusarium wilt resistance developed in Annigeri 1 genetic background (Mannur et al., 2019). Similarly, higher genome recovery in early generations is not uncommon. For instance, in the case of rice (*Oryza sativa* L.), 91.6% RPG recovery in the BC_2_F_1_ generation was reported while pyramiding blast resistance genes into an elite Basmati rice (Singh et al., 2013). In the case of ILs derived from Pusa 372 and DCP 92‐3, >90% RPG recovery was observed. Similarly, >90% background genome recovery was also reported in earlier studies in chickpea in progenies after three backcross generations (Pratap et al., 2017; Varshney et al., 2013a, 2014b).

Pusa 372 is a popular and widely adapted landmark chickpea variety released for the northwestern plains zone, the northeaster plains zone, and the central zone in India. A total of 20 ILs were developed after three cycles of backcrossing of Pusa 372 with ICC 4958 followed by two selfings. Interestingly, all introgression lines outperformed both donor and recipient parents evaluated under rainfed conditions at ICAR–IARI. Large variation for DM was observed among the ILs. The early flowering and early maturing ILs identified in the present study will fit well in double cropping, therefore, these ILs are ideal for the sustainability of rice‐based cropping systems. Further, because of short duration they also fit into cropping systems of the central zone. Pusa 362 is a bold‐seeded and Fusarium wilt resistant variety released for cultivation in the northwestern plains zone in India by ICAR–IARI in 1995. A total of 20 ILs were generated after two cycles of backcrossing with Pusa 362 and two rounds of selfing. These ILs have shown significant variation in RDW and RLD. In an earlier study, 257 recombinant inbred lines derived from a cross between ICC 4958 (large root system) and ‘Annigeri’ (an agronomically elite variety) also showed variation for RLD at 35 d after sowing in field conditions under terminal drought (Serraj et al., 2004). Lynch and Wojciechowski (2015) have also considered RL and root depth as important root architectural traits that directly influence the acquisition of water and nutrients from the soil. Most of the ILs are expected to be phenotypically similar to each other and to the recurrent parent Pusa 362. However, significant variation among the ILs was observed for seed yield and PPP. Some studies have reported that yield potential is known to contribute to yield under water stress in several crops including chickpea (Pang et al., 2017; Pushpavalli et al., 2020; Varshney et al., 2018). The recurrent parent Pusa 362 yielded 521.33 g while the donor parent ICC 4958 gave a yield of 276 g. Two ILs (BG 3097 and BG 4005), which gave significantly higher yield than Pusa 362 under rainfed condition, were nominated for multilocation testing and future release under ICAR–AICRP on Chickpea.

DCP 92‐3 is a lodging‐ and wilt‐resistant variety with yellowish brown, medium bold seeds released by IIPR in 1997 for cultivation in Punjab, Haryana, Delhi, northern Rajasthan, and western Uttar Pradesh. A total 21 ILs (BC_3_F_3_ line) were developed by MABC of DCP 92‐3 with ICC 4958 after involving three cycles of backcrossing and two rounds of selfing. Based on 2 yr of yield evaluation, IPC(L4‐14), IPC(L4‐16), and IPC(L19‐1) ILs with >16% yield increase over the recurrent parent were nominated for AICRP Chickpea trials. IPC(L4‐14) with 11.0% yield over check and the recurrent parent in AVT1 trial has been promoted to AVT2 trial.

Besides developing superior lines with enhanced yield under rainfed or drought‐stress condition through introgression of the *QTL‐hotspot* into different genetic backgrounds, we also report the successful release of one improved variety, Pusa Chickpea 10216, in India (https://icar.org.in/content/development-two-superior-chickpea-varieties-genomics-assisted-breeding). Pusa Chickpea 10216 recorded an overall weighted mean yield advantage of 16% over recurrent parent Pusa 372 across all the centers tested in national DTIL trials under AICRIP over two consecutive years, 2017–2018 (8% over Pusa 372 over six locations) and crop season 2018–2019 (30% over Pusa 372 over five locations). It is a profusely branching variety with more pods per unit area with overall weighted mean yield of 1,475 kg ha^−1^ and has yield potential of 2,575 kg ha^−1^ under drought stress over the recurrent parent Pusa 372, which yielded 1,272 kg ha^−1^. It is an early flowering (50–55 d) and early maturing variety (106 d) and fits in central and southern zones. Further, it is moderately resistant to Fusarium wilt, dry root rot, and pod borer.

In summary, the present study demonstrates that the introgression of the *QTL‐hotspot* into three elite genetic backgrounds enhances drought tolerance and seed yield under drought stress conditions. Further, superior ILs developed in different genetic backgrounds can be tested for possible release as improved varieties. In addition to developing the superior lines, we also reported the release of improved varieties with enhanced drought tolerance and higher yield under drought stress.

## AUTHOR CONTRIBUTIONS

C.B., S.T., K.R.S. supervised the crossing and selection of lines in breeding program. M.T. supervised the marker genotyping and data analysis for selection of lines. R.K.S., S.S. conducted the experiments. M.T., M.R., B.S.P., A.C., R.P., Y.T., B.M., P.S.S., A.K.S., S.K.C., G.P.D., N.P.S., R.K.V. contributed resources, data analysis and interpretation. A.R., A.V. did the statistical analysis. R.K.V. conceived the idea and provided the technical support and guidance to the overall research. All authors read and approved the MS.

## CONFLICT OF INTEREST

The authors declare that the research was conducted in the absence of any commercial or financial relationships that could be construed as a potential conflict of interest.

## Supporting information

Supplemental Table S1. Summary of marker polymorphism between the donor and recipient parents chosen for MABC program
Supplemental Table S2. Summary of markers used for background selection in three combinations (Pusa 372 × ICC 4958, Pusa 362 × ICC 4958 and DCP 92‐3 × ICC 4958)
Supplemental Table S3. ANOVA for agronomic and yield‐related traits evaluated on BC3F3 lines developed in the background of Pusa 372
Supplemental Table S4. Mean performance of 20 BC3F3 lines for different traits in the genetic background of Pusa 372
Supplemental Table S5. ANOVA for agronomic and yield‐related traits evaluated on BC2F3 lines developed in the background of Pusa 362
Supplemental Table S6. Mean performance of introgression lines for different root related traits under rainfed conditions developed in Pusa 362 background|
Supplemental Table S7. ANOVA for agronomic and yield‐related traits evaluated on BC2F3 lines developed in the background of Pusa 362
Supplemental Table S8. Mean performance of introgression lines for different traits in the background of Pusa 362
Supplemental Table S9. ANOVA for morphological and yield‐related traits evaluated on BC3F3 lines developed in the background of DCP 92‐3
Supplemental Table S10. Mean performance of 20 BC3F3 for morphological and yield‐related traits developed in DCP 92‐3 background
