# Supplementary material for: Introgression of “QTL‐hotspot” region enhances drought tolerance and grain yield in three elite chickpea cultivars
Source: Plant Genome. 2021 Jan 22;14(1):e20076. doi: 10.1002/tpg2.20076 (PMC12807433; doi:10.1002/tpg2.20076)
Supplement: Supplementary file 1 [file TPG2-14-e20076-s001.pdf]

Supplementary Table S1. Summary of marker polymorphism between the donor and recipient parents chosen for MABC program

SSR markers in the "*QTL-hotspot*" region	Cross combinations		
	**Pusa 372 × ICC 4958**	**Pusa 362 × ICC 4958**	**DCP 92-3 × ICC 4958**
TAA170	M	M	P
ICCM0249	M	M	M
GA24	M	M	M
STMS11	M	M	M
GA11	M	M	M
NCPGR21	P	P	P
NCPGR127	P	P	M
TR11	M	M	P

P= Polymorhic, M=Monomorphic

Supplementary Table S2. Summary of markers used for background selection in three combinations (Pusa 372 × ICC 4958, Pusa 362 × ICC 4958 and DCP 92-3 × ICC 4958)

Marker name	Linkage group	Pusa 372 × ICC 4958	Pusa 362 × ICC 4958	DCP 92-3 × ICC 4958
NCPGR184	CaLG01	P	P	M
TA113	CaLG01	P	M	P
TA203	CaLG01	P	M	M
NCPGR117	CaLG01	P	P	M
NCGR263	CaLG01	P	M	P
TR43	CaLG01	P	P	M
GAA40	CaLG01	M	P	M
ICCM0297	CaLG01	M	P	M
H1G16	CaLG01	M	P	M
TA122	CaLG01	M	M	P
TA30	CaLG01	M	M	P
TA47	CaLG01	M	M	P
NCPGR74	CaLG01	M	M	P
GA20	CaLG02	P	M	M
TAA194	CaLG02	P	P	M
H2110	CaLG02	P	M	M
TAA60	CaLG02	P	P	M
GA16	CaLG02	P	M	M
CaSTMS23	CaLG02	P	M	M
CaM0111	CaLG02	M	P	M
TR19	CaLG02	M	P	P
TS82	CaLG02	M	P	M
TA96	CaLG02	M	P	M
H1F05	CaLG02	M	P	M
TA103	CaLG02	M	P	M
STMS12	CaLG02	M	M	P
TA110	CaLG02	M	M	P
NCPGR173	CaLG03	P	P	M
TA135	CaLG03	P	P	M
TS10	CaLG03	P	M	P
NCPGR100	CaLG03	P	M	M
NCPGR103	CaLG03	P	M	M
GAA47	CaLG03	P	M	M
H1F14	CaLG03	M	P	M
H4H06	CaLG03	M	P	M
TA64	CaLG03	M	P	M
TR26	CaLG03	M	P	M

NCPGR141	CaLG03	M	M	P
NCPGR170	CaLG03	M	M	P
TA18	CaLG03	M	M	P
NCPGR12	CaLG03	M	M	P
NCPGR91	CaLG04	P	M	M
TR11	CaLG04	P	M	M
ICCM257	CaLG04	P	M	M
TA2	CaLG04	P	P	M
TA22	CaLG04	P	M	M
TR56	CaLG04	M	P	P
TA34	CaLG04	M	P	M
TS54	CaLG04	M	P	M
H1B17	CaLG04	M	P	M
CaM2049	CaLG04	M	P	M
CaM0480	CaLG04	M	P	M
CaM0446	CaLG04	M	P	M
CaM0923	CaLG04	M	P	M
TS72	CaLG04	M	P	M
CaM0845	CaLG04	M	P	M
NCPGR203	CaLG04	M	M	P
NCPGR51	CaLG04	M	M	P
TA89	CaLG04	M	M	P
H1B17	CaLG04	M	M	P
NCPGR254	CaLG05	P	M	M
TR18	CaLG05	P	M	M
H1H07	CaLG05	P	M	M
NCPGR81	CaLG05	P	M	M
H1A18	CaLG05	P	M	P
NCPGR228	CaLG05	P	M	M
TS35	CaLG05	M	P	M
ICCM76	CaLG05	M	P	M
CaM0038	CaLG05	M	P	M
CaM0639	CaLG05	M	P	M
CaM1068	CaLG05	M	P	M
NCPGR232	CaLG05	M	M	P
TAA58	CaLG05	M	M	P
TAASH	CaLG05	M	M	P
TS43	CaLG05	M	M	P
NCPGR37	CaLG05	M	M	P
TA11	CaLG05	M	M	P
TA127	CaLG05	M	M	P

TA39	CaLG05	M	M	P
NCPGR87	CaLG06	P	M	M
GA26	CaLG06	P	P	M
GA21	CaLG06	P	M	M
NCPGR76	CaLG06	P	M	M
NCPGR79	CaLG06	P	M	M
H3C08	CaLG06	P	M	M
TA106	CaLG06	M	P	M
GA34	CaLG06	M	P	M
TA14	CaLG06	M	P	P
CaM0464	CaLG06	M	P	M
TA22	CaLG06	M	P	M
CaM1402	CaLG06	M	P	M
TA176	CaLG06	M	P	M
NCPGR153	CaLG06	M	M	P
NCPGR200	CaLG06	M	M	P
NCPGR202	CaLG06	M	M	P
NCPGR78	CaLG06	M	M	P
TA120	CaLG06	M	M	P
TA80	CaLG06	M	M	P
TA180	CaLG07	P	P	M
TA117	CaLG07	P	M	M
NCPGR130	CaLG07	P	M	M
NCPGR249	CaLG07	P	M	M
TAA55	CaLG07	P	M	M
TA142	CaLG07	P	M	M
TA116	CaLG07	M	P	M
H3A07	CaLG07	M	P	M
TA78	CaLG07	M	P	M
CaM0317	CaLG07	M	P	M
H1A06	CaLG07	M	M	P
NCPGR149	CaLG07	M	M	P
STMS25	CaLG07	M	M	P
TA179	CaLG07	M	M	P
NCPGR19	CaLG07	M	M	P
TS46	CaLG07	M	M	P
TA44	CaLG07	M	M	P
NCPGR89	CaLG08	P	M	M
TS57	CaLG08	P	M	M
H3C11	CaLG08	P	M	M
NCPGR138	CaLG08	P	M	M

NCPGR209	CaLG08	P	M	M
TA118	CaLG08	P	M	M
TR5	CaLG08	M	P	M
H1H14	CaLG08	M	P	M
H1D24	CaLG08	M	P	M
TA3	CaLG08	M	P	M
H1B04	CaLG08	M	M	P
H1B09	CaLG08	M	M	P
NCPGR264	CaLG08	M	M	P
NCPGR50	CaLG08	M	M	P
**Total markers used**		**47**	**53**	**46**

Supplementary Table S3. ANOVA for agronomic and yield related traits evaluated on BC_3F_3 lines developed in the background of Pusa 372

Source of variation	Degrees of freedom	Plant height (cm)	Days to 50% flowering	Days to maturity	Pods per plant	100 seed weight (g)	Seed yield (g)/ plot
**Replication**	2	8.32	4.16	0.44	0.29	1.21	20.44
**MABC lines**	21	6.12	3.59	1.56**	1.23**	7.32	12662.7**
**Error**	42	5.18	2.51	0.41	0.31	5.79	31.32
**CV**		1.86	0.40	0.58	6.02	2.18	4.56

*Significant at 0.01 probability level

Supplementary Table S4. Mean performance of 20 BC_3F_3 lines for different traits in the genetic background of Pusa 372

Introgression line	Plant height (cm)	Days to 50% flowering	Days to Maturity	Pods per plant	100 seed weight (gm)	Yield per plot (gm)	Increase in yield over recurrent parent (%)
Pusa 372_IL01	47.67	62	112	176	24.32	344.98	15
Pusa 372_IL02	41.33	64	113	149	24.03	342.14	14
Pusa 372_IL03	43.67	66	118	187	22.01	338.15	13
Pusa 372_IL04	47.00	64	118	179	21.96	362.12	21
Pusa 372_IL05	54.67	62	120	169	20.19	339.53	13
Pusa 372_IL06	54.33	68	112	170	22.21	356.16	19
Pusa 372_IL07	49.33	68	113	196	23.07	348.19	16
Pusa 372_IL08	51.33	67	121	208	20.69	350.88	17
Pusa 372_IL09	53.67	66	114	158	24.12	348.69	16
Pusa 372_IL10	47.67	74	112	198	21.12	345.22	15
Pusa 372_IL11	54.00	74	111	175	23.11	338.42	13
Pusa 372_IL12	62.00	68	126	180	24.02	366.28	22
Pusa 372_IL13	58.67	68	110	181	21.97	364.72	21
Pusa 372_IL14	58.33	67	121	227	20.49	344.62	15
Pusa 372_IL15	54.33	64	105	205	21.53	339.51	13
Pusa 372_IL16	59.33	69	108	201	22.18	328.92	10
Pusa 372_IL17	54.00	68	116	185	22.56	366.34	22
Pusa 372_IL18	58.67	74	119	195	21.75	364.21	21
Pusa 372_IL19	55.67	66	118	180	21.39	342.56	14
Pusa 372_IL20	54.33	67	122	150	22.47	341.29	14
**ICC 4958 (Donor parent)**	**47.32**	**83.12**	**117.00**	**52.18**	**27.46**	**221.32**	
**Pusa 372 (Recurrent parent)**	**50.67**	**74**	**122**	**187**	**17.32**	**300.21**	
**Minimum**	**41.33**	**62**	**105**	**52.18**	**17.32**	**221.32**	
**Maximum**	**62.00**	**83.12**	**126**	**227**	**27.46**	**366.34**	
**Mean**	**52.64**	**68.32**	**115.82**	**177.64**	**22.27**	**340.66**	

Supplementary Table S5. ANOVA for agronomic and yield related traits evaluated on BC_2F_3 lines developed in the background of Pusa 362

Source of variation	Degrees of freedom	Mean sum of squares							
		Taproot length (TRL; cm)	Total root length (RL; cm)	Root surface area (RA; cm^2)	Root volume (RV; cm^3)	Average root diameter (RD; mm)	Root dry weight (RDW; gm)	Specific root length (SRL; cm g^{-1})	Root tissue density (RTD; g cm^{-3})
Replication	2	0.92	3355.23**	389.28**	0.1955**	0.06117	0.1379	781.49	0.2607
Introgression lines	21	21.1*	2066.8**	90.4**	0.0839**	0.02869	0.1093**	4183.94**	0.2187
Error	42	11.63	473.53	28.02	0.0229	0.02139	0.0463	1120.34	0.1501

*Significant at 0.05; **Significant at 0.01

Supplementary Table S6. Mean performance of introgression lines for different root related traits under rainfed conditions developed in Pusa 362 background

Introgression line	Tap root length (TRL; cm)	Total root length (RL; cm)	Root surface area (RA; cm^{2})	Root volume (RV; cm^3)	Average root diameter (RD; mm)	Root dry weight (RDW; gm)	Specific root length (SRL; cm g-1)	Root tissue density (RTD; g cm-3)
Pusa 362_IL01	35	117	33.89	0.846	0.975	0.863	136.88	1.04
Pusa 362_IL02	27.67	119.3	35	0.958	0.89	0.92	132.62	0.966
Pusa 362_IL03	33.67	116.25	35.65	0.803	0.88	0.957	122.66	1.23
Pusa 362_IL04	33.67	124.25	43.71	1.21	0.991	1.14	114.6	0.965
Pusa 362_IL05	30.67	175.47	32.96	0.706	1.08	0.987	177	1.42
Pusa 362_IL06	31.67	121.8	42.73	1.08	0.918	1.4	88.59	1.34
Pusa 362_IL07	34	128.03	35.31	0.867	0.787	0.927	140.84	1.08
Pusa 362_IL08	33	190.7	42.45	0.935	0.787	1.09	172.04	1.17
Pusa 362_IL09	31.33	102.17	33.72	0.763	0.877	1.15	96.58	1.53
Pusa 362_IL10	33	134.27	37.39	0.838	0.847	0.85	162.42	1.04
Pusa 362_IL11	28.66	117.88	28.73	0.92	0.887	0.82	145.95	0.92
Pusa 362_IL12	28.66	112.18	34.1	0.835	0.943	0.923	135.7	1.2
Pusa 362_IL13	27.33	111.13	29.16	0.49	0.843	1.02	109.06	2.19
Pusa 362_IL14	33.33	145.37	39.34	0.97	1.01	0.953	155	1.01
Pusa 362_IL15	28	113.75	33.72	0.82	0.847	0.933	121.73	1.16
Pusa 362_IL16	30	105.45	33.25	0.916	1.15	1.1	98.86	1.2
Pusa 362_IL17	31	102.8	28.8	0.823	0.968	0.92	114.12	1.11
Pusa 362_IL18	32	109.4	29.73	0.451	0.788	0.45	244.99	1.02
Pusa 362_IL19	31.33	189.18	44	0.86	0.825	0.993	205.64	1.27
Pusa 362_IL20	31.33	129.83	24.78	0.78	0.948	0.793	164	1.01
**ICC4958 (Donor parent)**	**36.33**	**140.83**	**41.3**	**0.98**	**1.02**	**1.27**	**112.55**	**1.31**
**Pusa 362 (Recurrent parent)**	**26.33**	**102.53**	**28.78**	**0.71**	**0.851**	**0.827**	**124.14**	**1.2**
**Minimum**	**26.33**	**102.17**	**24.78**	**0.451**	**0.787**	**0.45**	**88.59**	**0.92**
**Maximum**	**36.33**	**190.7**	**44**	**1.21**	**1.15**	**1.4**	**244.99**	**2.19**
**Mean**	**31.3**	**128.31**	**34.92**	**0.84**	**0.91**	**0.96**	**141.9**	**1.19**

Supplementary Table S7. ANOVA for agronomic and yield related traits evaluated on BC_2F_3 lines developed in the background of Pusa 362

Source of variation	Degrees of freedom	Mean Square							
		Days to 50% flowering	Days to maturity	Pods per plant	Plant height (cm)	100 seed weight (gm)	Seed yield (gm)	Biomass (gm)	Harvest index (%)
**Replications**	2	3.47	1.20	0.15	14.83	0.65	13136.61	3022.25	0.01
**Introgression lines**	21	3.92	2.44**	0.77**	8.69	9.88	5480.77	19956.93**	0.01
**Introgression lines Vs Parents**	1	14.55	3.21	2.51**	2.28	51.06**	7030.18	16299.62	0.04**
**Error**	42	2.23	0.86	0.22	7.01	5.73	4954.87	9300.87	0.01

*Significant at P<0.05; **Significant at P<0.01

Supplementary Table S8. Mean performance of introgression lines for different traits in the background of Pusa 362

Introgression line	Plant height (cm)	Days to 50% flowering	Days to maturity	Pods/plant	Biomass (gm)	Harvest index (%)	100 Seed weight (gm)	Seed yield (gm)
Pus 362_IL01	47.67	80.00	117.33	46.83	893.33	0.46	24.87	409.25
Pus 362_IL02	49.33	80.00	117.33	34.99	853.33	0.37	24.93	249.01
Pus 362_IL03	46.87	77.00	115.00	34.10	860.00	0.43	23.19	369.59
Pus 362_IL04	47.93	79.00	117.33	53.13	855.85	0.46	24.53	426.15
Pus 362_IL05	49.67	80.00	118.00	48.37	1040.85	0.43	24.93	427.08
Pus 362_IL06	47.13	77.00	116.67	50.09	1038.37	0.45	26.68	426.72
Pus 362_IL07	48.93	80.00	116.67	38.95	861.67	0.42	24.09	386.86
Pus 362_IL08	47.27	80.00	116.67	36.04	788.33	0.45	22.96	359.14
Pus 362_IL09	46.47	79.00	118.00	41.41	826.67	0.44	24.87	360.39
Pus 362_IL10	48.13	80.00	118.00	42.29	871.67	0.45	29.19	391.99
Pus 362_IL11	46.93	79.00	115.00	42.49	720.00	0.48	24.51	346.14
Pus 362_IL12	45.80	79.00	117.33	50.01	737.00	0.53	23.81	371.42
Pus 362_IL13	51.13	79.00	118.00	41.32	860.00	0.49	25.88	414.96
Pus 362_IL14	46.80	80.00	118.00	34.89	775.78	0.44	26.17	393.91
Pus 362_IL15	47.87	80.00	118.00	33.89	838.33	0.41	23.65	345.42
Pus 362_IL16	46.27	79.00	116.67	31.33	840.00	0.42	24.59	353.31
Pus 362_IL17	45.20	79.00	118.00	42.03	816.67	0.48	23.03	325.82
Pus 362_IL18	46.13	77.00	117.33	35.43	843.28	0.43	26.51	351.23
Pus 362_IL19	46.93	80.00	118.00	38.73	896.67	0.41	24.07	367.52
Pus 362_IL20	48.60	80.00	117.33	47.69	810.00	0.44	24.23	354.77
**ICC 4958**	**45.00**	**81.67**	**118.00**	**52.18**	**768.35**	**0.35**	**30.17**	**294.09**
**Pusa 362**	**51.40**	**80.00**	**118.00**	**48.09**	**1056.67**	**0.36**	**25.61**	**377.00**
**Minimum**	**45.20**	**77.00**	**115.00**	**31.33**	**720.00**	**0.37**	**22.96**	**249.01**
**Maximum**	**51.13**	**80.00**	**118.00**	**53.13**	**1040.85**	**0.53**	**29.19**	**427.08**
**Mean**	**47.50**	**79.16**	**117.23**	**40.86**	**853.57**	**0.44**	**24.87**	**372.42**

Supplementary Table S9. ANOVA for morphological and yield related traits evaluated on BC_3F_3 lines developed in the background of DCP 92-3

Source of variation	Df	Plant height (cm)	Primary branches	Secondary branches	Tertiary Branches	Days to 50% flowering	Days to maturity	Yield per m^2	Pods per plant	100 seed weight (gm)
**Replications**	2	39.1884	0.0177034	0.0741019	0.0834048	1.144928	0.623188	3101.609	98.44928	1.6401449
**Introgression lines**	22	91.94**	0.022323	0.21*	1.27**	61.68**	215.24**	1265**	2854.54**	17.354941
**Error**	44	13.1278	0.025828	0.0909348	0.3439341	10.220685	11.19895	6256.791	650.6311	0.5871146
**CV %**		7.97712	11.18951	12.41157	12.3749	5.3685	2.804315	11.31052	24.49228	4.499587

*Significant at P<0.05; **Significant at p<0.01

Supplementary Table S10. Mean performance of 20 BC_3F_3 for morphological and yield related traits developed in DCP92-3 background

Introgression line	Plant height (cm)	Primary Branches	Secondary branches	Tertiary branches	Days to 50% flowering	Days to maturity	Pods per plant	100 seed weight (gm)	Yield per plot (gm)
IPC16(7)	51.33	2.00	6.32	23.94	57.00	128.00	112.33	15.33	774.00
IPC19(1)	41.33	1.63	4.28	16.01	59.33	111.67	58.67	13.80	877.00
IPC21(7)-1	53.33	1.63	5.55	19.63	55.33	116.67	85.00	15.10	914.00
IPC21(7)-2	50.00	2.00	6.27	19.29	68.67	125.00	100.00	14.20	772.00
IPC22(33)-2	50.00	2.21	9.24	28.96	61.00	121.67	144.67	15.30	900.00
IPC4(11)	56.00	2.00	6.17	17.14	57.33	124.00	74.00	14.40	435.00
IPC4(13)	43.33	2.00	5.66	25.34	71.33	107.67	99.00	17.67	748.00
IPC4(14)	48.00	2.31	6.66	22.69	57.00	97.67	91.33	21.00	1120.00
IPC4(16)	44.67	2.31	6.61	30.09	61.33	109.00	118.33	19.07	808.00
IPC4(18)	46.00	2.00	5.66	19.83	63.00	122.00	144.33	19.10	545.00
IPC4(2)	50.33	2.00	3.65	20.72	61.33	127.00	137.33	18.43	343.00
IPC4(20)	41.33	2.31	7.38	21.50	66.00	131.00	129.00	18.00	658.00
IPC4(21)	35.33	2.31	6.00	21.90	55.67	129.00	119.00	17.93	739.00
IPC4(22)	43.00	2.00	5.29	30.01	65.00	115.67	133.33	19.00	725.00
IPC4(24)	42.00	2.00	6.61	34.52	58.00	117.33	142.33	18.03	370.00
IPC4(25)	48.00	2.00	5.91	17.66	58.00	124.00	79.33	16.43	982.00
IPC4(26)	44.67	2.31	6.27	16.97	56.00	114.00	96.00	16.77	360.00
IPC4(5)	53.67	2.00	5.97	22.14	55.00	123.33	111.00	18.40	760.00
IPC4(7)	42.00	2.31	8.31	32.17	57.00	111.33	155.00	17.27	772.00
IPC7(20)	37.67	2.00	4.28	15.31	56.00	123.33	80.67	14.87	695.00
IPC9(5)	38.67	2.00	5.29	14.64	59.00	132.00	74.33	13.47	748.00
**DCP 92-3 (Recurrent parent)**	**39.00**	**39.00**	**39.00**	**39.00**	**39.00**	**39.00**	**39.00**	**39.00**	**39.00**

**ICC 4958 (Donor parent)**	**45.00**	**45.00**	**45.00**	**45.00**	**45.00**	**45.00**	**45.00**	**45.00**	**45.00**
**Minimum**	**35.33**	**1.63**	**3.65**	**14.64**	**55.00**	**97.67**	**58.67**	**13.47**	**343.00**
**Maximum**	**56.00**	**2.31**	**9.24**	**34.52**	**71.33**	**132.00**	**155.00**	**21.00**	**1120.00**
**Average**	**45.75**	**2.06**	**6.06**	**22.40**	**59.92**	**119.59**	**108.81**	**16.84**	**716.43**
